# Carotenoids in Paprika Fruits and Ajvar: Chemical Characterization and Biological Activity

**DOI:** 10.3390/foods14060914

**Published:** 2025-03-07

**Authors:** Stefan Kolašinac, Ilinka Pećinar, Mirjana Cvetković, Dejan Gođevac, Nemanja Stanisavljević, Mile Veljović, Ivan Šoštarić, Svetlana Aćić, Dragana Rančić, Marina Mačukanović-Jocić, Jelena Kolašinac, Zora Dajić Stevanović

**Affiliations:** 1 Department of Agrobotany, Faculty of Agriculture, University of Belgrade, Nemanjina 6, 11080 Belgrade, Serbia; ilinka@agrif.bg.ac.rs (I.P.); sostaric@agrif.bg.ac.rs (I.Š.); acic@agrif.bg.ac.rs (S.A.); rancicd@agrif.bg.ac.rs (D.R.); marmajo@agrif.bg.ac.rs (M.M.-J.); 2 Institute of Chemistry, Technology and Metallurgy, National Institute of the Republic of Serbia, University of Belgrade, Njegoševa 12, 11000 Belgrade, Serbia; mirjana.cvetkovic@ihtm.bg.ac.rs (M.C.); dgodjev@chem.bg.ac.rs (D.G.); 3 Institute of Molecular Genetics and Genetic Engineering, University of Belgrade, 11010 Belgrade, Serbia; nemanja.stanisavljevic@imgge.bg.ac.rs; 4 Department of Food Technology and Biochemistry, Faculty of Agriculture, University of Belgrade, Nemanjina 6, 11080 Belgrade, Serbia; mile.veljovic@agrif.bg.ac.rs (M.V.); jelenaotasevic@hotmail.rs (J.K.)

**Keywords:** carotenoids, paprika, in vitro digestion, beta-carotene, Raman spectroscopy, HPTLC

## Abstract

In this study, carotenoids from four different paprika genotypes were analyzed at various maturation stages, as well as in Ajvar, a traditional Balkan product made from fully matured roasted paprika fruits. For this purpose, the HPTLC analytical method was used, and five dominant carotenoids were analyzed: β-carotene, lutein, zeaxanthin, capsanthin, and β-cryptoxanthin. Additionally, total carotenoids were analyzed spectrophotometrically, antioxidant capacity was determined, and their bioavailability was assayed using in vitro digestion. Finally, Raman spectroscopy, a non-destructive analytical method, was used to estimate the total carotenoid content. The results showed that the amount of all investigated carotenoids is the highest in the final maturity stage (0.38 g/100 g DM to 1.55 g/100 g DM). On the other hand, the lowest concentration of all investigated carotenoids was detected at the first stage of maturation, ranging from 0.01 g/100 g DM to 0.25 g/100 g DM. However, the analysis of carotenoid content in Ajvar showed a tendency for a decrease in concentration compared to their quantity in fresh fruits, although this was also dependent on the genotype (1.9–66.98% according to HPTLC results and 16.14–82.36% according to spectrophotometry). Antioxidant tests indicated an increase in antioxidant capacity with the ripening of paprika fruits, confirming the role of carotenoids as compounds capable of neutralizing harmful oxygen species (DPPH ranged from 0.21 to 1.50 µmol/g TEAC, CUPRAC ranged from 0.185 to 0.297 mg AsA/g DM, FRP ranged from 9.33 to 25.66 mg AsA/g DM). Quantification of total carotenoids by Raman spectroscopy showed that results were highly correlated with those obtained by HPTLC and the spectrophotometric method, highlighting the potential of Raman spectroscopy for carotenoid quantification. Based on the obtained results, it can be concluded that the traditional product Ajvar represents an important source of carotenoids, which are preserved after heat treatment with high biological activity relative to the final ripening stage of the paprika. Furthermore, the bioavailability of carotenoids from Ajvar is significantly higher compared to the results from fresh paprika analysis.

## 1. Introduction

Paprika (*Capsicum annuum* L.) belongs to the family Solanaceae and is one of the most cultivated and utilized vegetable species [1]. The genus *Capsicum* consists of 22 wild and 5 domesticated species: *C. annuum* L., *C. baccatum* L., *C. chinense* Jacq., *C. frutescens* L., and *C. pubescens* R. and P.

The fruit of paprika can vary in color, shape, and size both between and within species. The color of mature fruits ranges from white to dark red. From the perspective of consumer preferences, color intensity, taste, aroma, and the level of pungency in certain species or genotypes are cited as the main quality attributes of the fruit. Paprika fruits from different genotypes, lines, and populations differ significantly in their chemical composition, including the amount of ascorbic acid (vitamin C), tocopherols, flavonoids, and carotenoids [2]. Carotenoids offer numerous physiological benefits. β-carotene, along with α-carotene and β-cryptoxanthin, serves as a source of provitamin A. Studies have demonstrated an inverse relationship between some chronic diseases and carotenoid intake through diet. Research has shown that carotenoids reduce oxidative stress, inhibit tumor cells, protect against cardiovascular diseases, and cataract formation [3,4].

*C. annuum* and *C. frutescens* (chili paprika) are the most commonly used paprika species in human nutrition worldwide. The fruits can be used fresh, dried, frozen, preserved, or processed and are an important source of carotenoid pigments due to their high concentration [5]. One of the most widely used products in the Balkans is Ajvar—made by roasted peppers (without seeds).

To date, high-performance liquid chromatography (HPLC) [2], high-performance thin-layer chromatography (HPTLC) [6], and Raman spectroscopy [7] are among the most frequently used techniques for carotenoid analysis. However, while HPTLC and Raman spectroscopy have primarily been employed for carotenoid detection in paprika samples, they have not yet been widely utilized for comprehensive qualitative and quantitative analysis.

This study aims to characterize the dominant and total carotenoids in selected paprika genotypes and the traditional paprika-based product, Ajvar, using HPTLC, Raman spectroscopy, and spectrophotometry. Specifically, the qualitative and quantitative analysis of key carotenoids, including β-carotene, β-cryptoxanthin, lutein, zeaxanthin, and capsanthin, was conducted by HPTLC. Additionally, the biological activity of the extracts was assessed through antioxidant activity tests and bioavailability studies using static in vitro gastrointestinal digestion. Since paprika fruits undergo thermal processing during Ajvar preparation, it is essential to compare the carotenoid content and biological activity between fresh fruits and the processed product. The integration of a static in vitro digestion model has further advanced the understanding of carotenoid bioavailability by simulating human gastrointestinal conditions to assess their stability, release, and absorption. The combination of these methodologies represents a comprehensive approach for analyzing carotenoids in paprika and paprika-based products, providing deeper insights into their composition, functionality, and nutritional impact. Notably, this is the first study to apply such a comprehensive analytical approach to Ajvar, integrating multiple advanced techniques to assess both its carotenoid profile and functional properties.

## 2. Materials and Methods

### 2.1. Plant Material

**Figure 1:**
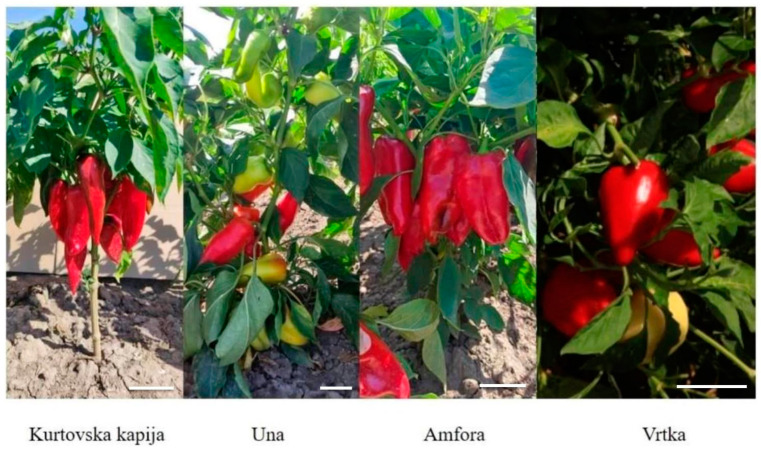
Investigated paprika genotypes in the field (white bar indicates 7 cm).

**Figure A1:**
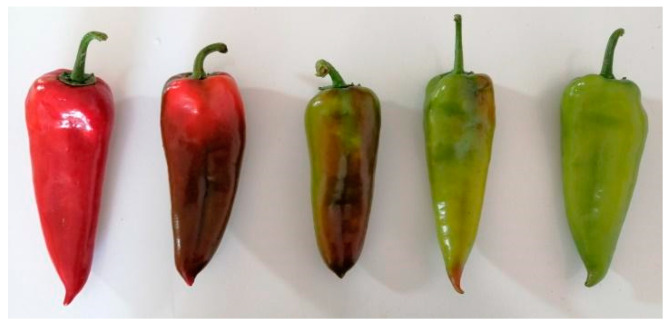
Kurtovska kapija genotype.

**Figure A2:**
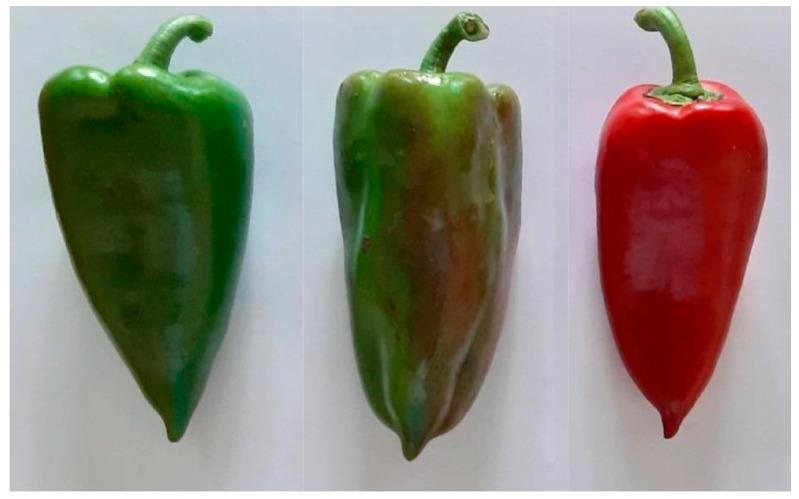
Amfora genotype.

**Figure A3:**
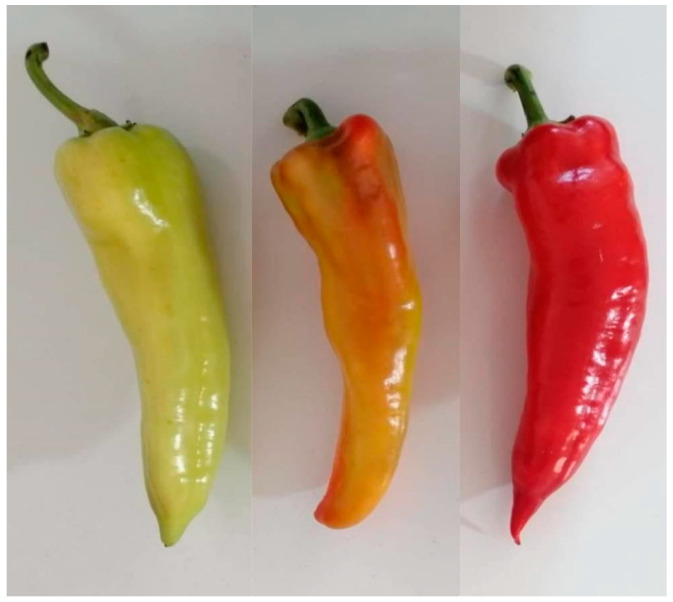
Una genotype.

**Figure A4:**
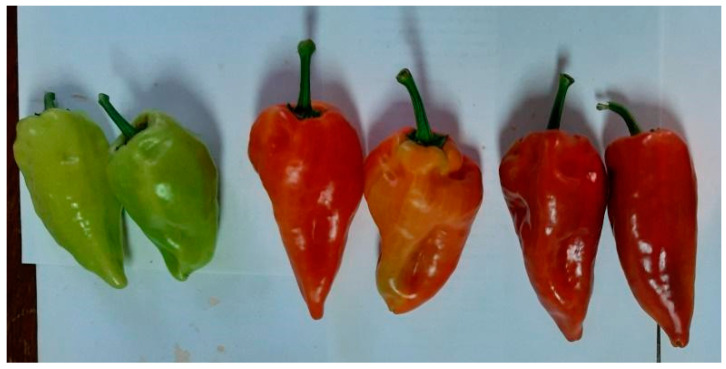
Vrtka genotype.

The fruits were sampled from experimental fields of the Institute of Field and Vegetable Crops in Novi Sad (45°20′ N; 19°51′ E), except for the Vrtka genotype, sampled from a production field in Bela Palanka (43°13′ N 22°19′ E), Republic of Serbia (Figure 1). The selected genotypes were Vrtka, Kurtovska kapija, Una, and Amfora. The paprika planting took place on 28 March 2019, in rows in an unheated greenhouse. Samples were taken in five successive developmental stages corresponding to different fruit colors: green (stage 1), green–brown (stage 2), brown–red (stage 3), red (stage 4), and dark red (stage 5). The genotype Kurtovska kapija was analyzed at all five maturity stages, while the other three genotypes were analyzed at three maturity stages (Figure A1, Figure A2, Figure A3 and Figure A4). A total of ten fruits were sampled per stage (one fruit per plant), resulting in 140 paprika fruits overall.

The fresh fruits (immediately after harvesting) were washed, cleaned of seeds, placenta, and stems, then ground and frozen at a temperature of −80 °C. Additionally, the Ajvar was also frozen. The frozen samples were subjected to freeze-drying (using a Christ ALPHA 2-4 LD plus freeze-dryer, Martin Christ Gefriertrocknungsanlagen GmbH, Osterode am Harz, Germany).

### 2.2. Preparation of Products from Analyzed Paprika Genotypes

**Figure 2:**
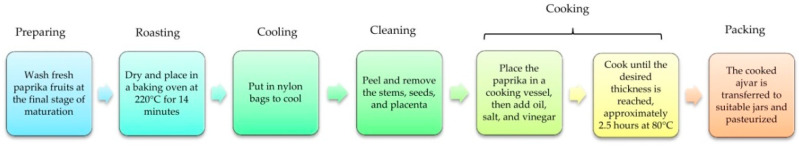
Flowchart of Ajvar production.

The traditional paprika-based product, Ajvar, was made from the aforementioned paprika genotypes at the stage of full physiological maturity under laboratory conditions at the Faculty of Agriculture, using a traditional recipe (Figure 2).

For 1 kg of Ajvar, the following ingredients are required:

- 2 kg of roasted paprika
- 100 mL of oil
- 20 g of salt
- 20 mL of 9% vinegar

**Figure 3:**
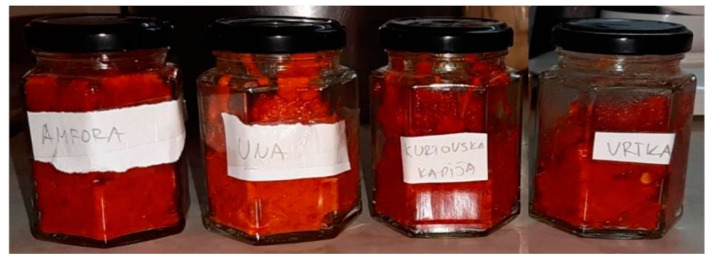
Ajvar produced from paprika in the physiological maturation stage; from left to right: Amfora, Una, Kurtovska kapija, and Vrtka genotypes.

The traditional Ajvar recipe is as follows:

Wash the paprika fruits, place them on a baking tray, and roast them in a preheated oven at 220 °C for 14 min. After roasting, let them cool in a nylon bag, and then peel them, removing the stems, seeds, and placenta. Once cleaned, transfer the paprikas to an appropriate cooking vessel, add the ingredients from the recipe, and cook until the desired thickness is achieved. The cooked Ajvar is then transferred to suitable jars and pasteurized (Figure 3). The detailed production process of Ajvar is presented in the work of Bogdanović et al. (2022) [8]. Additionally, the Ajvar was also frozen and freeze-dried.

### 2.3. Extraction of Carotenoids

The extraction of carotenoids from lyophilized paprika and Ajvar was carried out as follows: 5 g of the lyophilized sample was dissolved in 3 mL of a mixture of acetone and hexane in a 1:1 ratio. The prepared solutions were placed in an ultrasonic bath for 15 min and then centrifuged for 5 min at 13,500 rpm. The supernatant was used for further carotenoid analyses.

### 2.4. HPTLC Analysis

To determine the carotenoid composition of the tested extracts from paprikas and Ajvar, the technique of High Performance Thin Layer Chromatography (HPTLC) was applied. The paprika and Ajvar extracts were applied to a silica gel 60 F_254_ plate with dimensions of 20 × 10 cm (Merck—Darmstadt, Germany), which had been previously saturated with methanol and heated for 15 min at 120 °C (CAMAG TLC PLATE HEATER III). Then, 3 µL of the paprika/Ajvar extracts and 1 µL of the standard carotenoid mixture were applied to the plate using a semi-automatic TLC CAMAG Linomat 5 (Muttenz, Switzerland). A total of 19 spots were applied (17 samples and 2 standard mixtures). The solvent front was 70 mm, and the applied zone width was 7 mm. The initial position for applying the samples was ×10 mm (distance from the left edge of the plate) and Y 8 mm (distance from the bottom edge of the plate). Chromatographic separation was performed in a vertical chamber with dimensions of 20 × 10 cm (CAMAG, Muttenz, Switzerland) using a solvent mixture of hexane:acetone (8:2, *v*/*v*) with a volume of 120 mL for 15 min at 22 °C. Detection of the analyzed carotenoids was performed at a wavelength of 440 nm. The entire analysis was controlled using the WinCAT software (version 1.4.3.6336, CAMAG, Muttenz, Switzerland).

A series of carotenoid standards was prepared in the following concentrations: 0.085, 0.17, 0.22, and 0.33 mg/mL, except for β-cryptoxanthin, where the concentration range was 0.04 to 0.09 mg/mL.

After the development of the bands, the chromatographic plates were photographed (Nikon DIG Z30 LENS KIT W/12-28 DX PZ, Nikon, Tokyo, Japan), and the images were processed using the ImageJ software (ver. 1.43q Wayne Rasband, National Institutes of Health, Bethesda, MD, USA; https://imagej.net/ij/, accessed on 25 January 2025). Using this software, the image was separated into 3 channels (R—red, G—green, and B—blue), but for further analysis, only the B (blue) channel was used because it provided the best response.

The Kurtovska kapija genotype was analyzed in all 5 stages of ripening as a standard model genotype, while the genotypes Amfora, Una, and Vrtka were analyzed in the first, third, and fifth stages of ripening, which are considered critical developmental phases of the fruit. The traditional product, prepared in laboratory conditions from all the tested genotypes, was analyzed using the same methods.

Besides, the amount of total investigated carotenoid content (capsanthin + lutein + zeaxanthin + β-cryptoxanthin + β-carotene) (TICC) measured by HPTLC was conducted.

### 2.5. Determination of the Total Carotenoids Content (TCC)

Total carotenoids were determined spectrophotometrically according to the previously described method [9].

Procedure:

The obtained acetone–hexane extracts of paprika and Ajvar (supernatant) were directly measured at an absorbance of 450 nm. The amount of carotenoids is calculated according to the following formula:

μg/g of carotenoids = (A∙V∙106)/(E_1cm_∙100∙m) (1)

where:

A—absorbance of the sample at 450 nm;

V—total volume of extracts;

E_1cm_—extinction coefficient for the solvent used;

m—measured sample mass (in grams).

### 2.6. Raman Spectroscopy

Besides the above-mentioned, quantification of total carotenoids was conducted using the Raman spectroscopy method. For this purpose, Witec Alpha 300R (WITec Wissenschaftliche Instrumente und Technologie GmbH, Ulm, Germany) is equipped with a confocal Zeiss microscope (Zeiss, Jena, Germany). The instrument is equipped with 532 and 785 nm lasers. In this investigation, a 532 nm laser was used coupled with a 10× magnification objective. Raman scattering was detected by CCD (charge-coupled device) detector. Resolution was 3 cm^−1^. Spectral calibration was performed by checking the position of silicon (520.47 cm^−1^).

To form a model for quantification, β-carotene was chosen as the model molecule since it is the most abundant carotenoid in the fully ripe stage (stage 5). For the creation of the regression model, a series of standard β-carotene solutions were prepared in a mixture of acetone/hexane (1:1, *v*/*v*) with the following concentrations: 1320 ppm, 660 ppm, 330 ppm, 150 ppm, 50 ppm, 30 ppm, 20 ppm, 10 ppm, 5 ppm, and 2.5 ppm.

The samples used to test the model were Kurtovska kapija and Amfora at the fully mature physiological stage, as well as Ajvar made from the same genotypes. These genotypes were selected as model genotypes, as they are most commonly grown in our country for the purpose of obtaining high-quality raw material for making Ajvar. The samples and series of standard solutions were then transferred into quartz microcapillaries, after which Raman spectra were recorded.

To maintain the repeatability of the obtained spectra, the spectra were recorded three times over a period of 9 days (every third day) under identical conditions (laser wavelength, laser power, exposure time, temperature, and sample matrix). To prevent degradation of the standards and samples, the paprika extracts were stored at a temperature of −80 °C (ULT 250, Nordic Lab, Copenhagen, Denmark).

### 2.7. Determination of the Ability to Scavenge DPPH Radicals

DPPH• (1,1-diphenyl-2-picrylhydrazyl) represents a stable free radical that has an unpaired valence electron on a nitrogen bridge atom [10]. The “scavenging” of the DPPH radical is the basis of the DPPH test [11]. The method was first described in 1958 (Blois, 1958) [11] and later modified by many researchers. This test is based on the theory that a hydrogen donor is an antioxidant and measures the antioxidant capacity of a plant extract as a “scavenger” of 1,1-diphenyl-2-picrylhydrazyl (DPPH) radicals.

The mechanism of the reaction between carotenoids and the DPPH• radical can be explained by the following reaction:

CAROTENOID + DPPH• → CAROTENOID• + DPPH Purple color → Yellow color(2)

where the DPPH• is reduced to DPPH by accepting a hydrogen atom from the carotenoid molecule. During the reaction between the DPPH• radical and carotenoids, the color changes from purple to yellow [12].

The determination of the radical scavenging activity is based on the work of [13].

Procedure: 105 μL of the extract is mixed with 840 μL of a 150 μM DPPH• solution. The mixture is left to stand at room temperature in the dark for 30 min, after which the absorbance is measured at 515 nm. The percentage of DPPH• radical inhibition is calculated using the following formula:

% of inhibition = (Ab − As)/Ab∙100(3)

where:

Ab—blank absorbance;

As—absorbance of the plant extract.

The ability to scavenge free radicals is expressed as μmol Trolox equivalents per gram of dry weight of the sample (μmol/g dry weight of the sample).

### 2.8. Fe^3+^ Reduction Ability—FRP

The antioxidant activity of the extracts was also determined by the FRP method [14].

Procedure:

To a glass test tube, 0.5 mL of extract was added, mixed with 0.5 mL of 0.2 M sodium phosphate buffer (pH 6.6) and 0.5 mL of a 1% potassium ferricyanide solution. The mixture was then incubated in a water bath at 50 °C for 20 min. Afterward, 0.5 mL of 10% trichloroacetic acid solution was added, and the mixture was centrifuged (4000× *g*) for 5 min. The supernatant, 1.5 mL, was separated and mixed with 1.5 mL of distilled water and 0.3 mL of 0.1% ferric chloride solution. The blank contained all the reagents except for the paprika extract (water was added instead of the extract). Absorbance was measured at a wavelength of 700 nm. Ascorbic acid was used as a standard, and the results were expressed as mg of ascorbic acid (AsA) per g of dry weight.

### 2.9. Cupric Ion Reducing Antioxidant Capacity—CUPRAC

The determination of Cu^2+^ ion reduction ability—CUPRAC (Cupric Reducing Antioxidant Capacity)—was conducted using the method by [15].

Procedure:

In an Eppendorf tube, 0.350 mL of the original paprika/Ajvar extract (obtained supernatant) was added, followed by 0.350 mL of CuCl_2_, 0.350 mL of Neocuproine, and 0.350 mL of ammonium acetate buffer (pH 7). The samples were kept in the dark for 30 min, then centrifuged for 3 min, and the absorbance was read at 450 nm. The measurement was repeated three times for each sample, and the result was obtained as the average of the three measurements ± standard deviation, expressed in milligrams of ascorbic acid (AsA) equivalents per g of samples (mg AsA/g DM).

All methods for determining antioxidant capacity, as well as the total carotenoid content, were measured using a Shimadzu UV-1800 UV–vis spectrophotometer, Shimadzu, Kyoto, Japan.

### 2.10. In Vitro Digestion

The static in vitro digestion method was performed according to the method of [16]. For this analysis, the Una genotype and Ajvar made from the same genotype were used. The selection of the Una genotype was based on its lowest values of total carotenoids, particularly in stages 1 and 3, as well as the slower accumulation of key carotenoids in the fruit, such as β-carotene, β-cryptoxanthin, and capsanthin. The lyophilized samples (0.5 g) were rehydrated with 4.5 mL of distilled water and then subjected to the first stage of digestion, which involves the oral phase. To the rehydrated sample, the following were added: 3.5 mL of the simulated salivary fluid (SSF), 0.5 mL of salivary α-amylase solution (1500 U/mL), 25 µL of 0.3 M CaCl_2_, and 975 µL of water, shaken vigorously and incubated for 2 min at 37 °C.

The gastric digestion stage (food digestion in the stomach) was then performed by adding 7.5 mL of simulated gastric fluid (SGF), 1.6 mL of pepsin (25,000 U/mL), 5 µL of 0.3 M CaCl_2_, adjusting the pH to 3.0 with HCl, and adjusting the final volume to 20 mL with distilled water. The samples were then incubated for 2 h at 37 °C using an orbital shaker set to 300 rpm. After the incubation, the intestinal digestion stage began by adding 11 mL of simulated intestinal fluid (SIF), 5 mL of pancreatin (800 U/mL), 2.5 mL of bile salts (160 mM), 40 µL of 0.3 M CaCl_2_, adjusting the pH to 7.0 with NaOH, and the final volume of the mixture was adjusted to 40 mL with distilled water. The samples were incubated under the same conditions and duration as in the gastric phase. After the completion of the entire digestion process, the supernatants containing the bioavailable fraction were collected by centrifuging for 20 min at 4500× *g* using a centrifuge (5804R, Eppendorf, Hamburg, Germany), previously cooled to 4 °C. The collected supernatants were stored at −80 °C until analysis. The detailed composition of the saliva, gastric, and intestinal fluids is shown in Table 1. To determine the initial carotenoid content (before digestion), a hexane–acetone extract was used, as it was found to be the best extraction solvent. The total carotenoid content before and after digestion was measured spectrophotometrically using the method previously described.

TCC after simulated digestion was evaluated spectrophotometrically. Obtained results were compared with results of TCC of acetone/hexane extracts since this mixed solvent has shown the best level of carotenoid extraction.

### 2.11. Statistical Analysis

To compare differences in the amounts of individual carotenoids, total carotenoids, and antioxidant activity between the different genotypes and between ripening stages within the same genotype, a one-way analysis of variance (one-way ANOVA) was used. As a post hoc test, Duncan’s test was applied.

Results obtained by Raman spectroscopy were organized into a matrix consisting of 45 rows (objects) and 806 columns (variables–wavelengths). The spectra were processed with the aim of preserving the relationships of the obtained intensities using baseline correction. After that, a Multiple Linear Regression (MLR) model was applied to the entire spectra.

## 3. Results

### 3.1. Qualitative and Quantitative Analysis of Carotenoids from Paprika and Ajvar Extracts Using HPTLC Analytical Method

**Figure 4:**
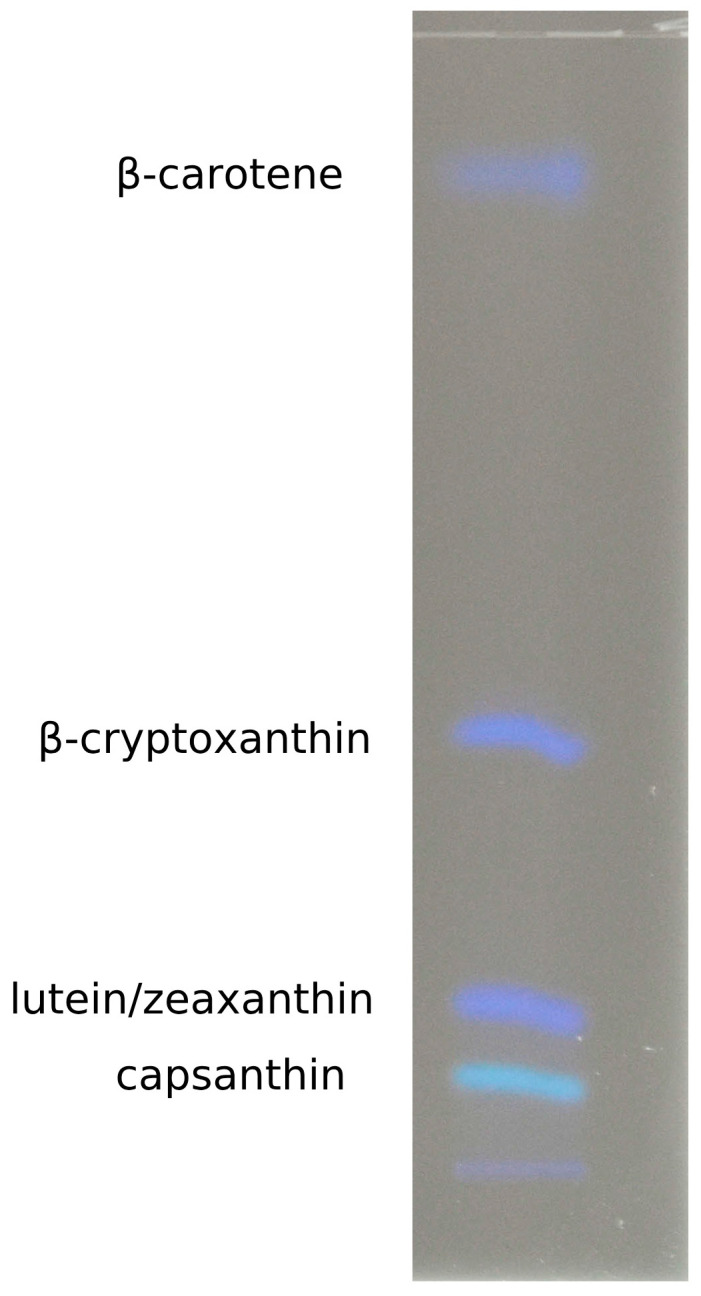
Bands of analytical standards separated on the TLC plate.

Prepared extracts were separated on TLC plates, and the presence of carotenoids was observed in all tested paprika genotypes, as well as in Ajvar. Analysis of the resulting bands, along with their comparison to standards, identified the presence of β-carotene, capsanthin, β-cryptoxanthin, lutein, and zeaxanthin. Since lutein and zeaxanthin have very similar Rf (retention factor) values, their results were analyzed together on this occasion (Figure 4). The Rf values for the standards of β-carotene, β-cryptoxanthin, lutein/zeaxanthin, and capsanthin were 0.87, 0.497, 0.111, and 0.054, respectively. Qualitative analysis was performed by comparing the tested samples with the Rf values of the standards.

**Figure 5:**
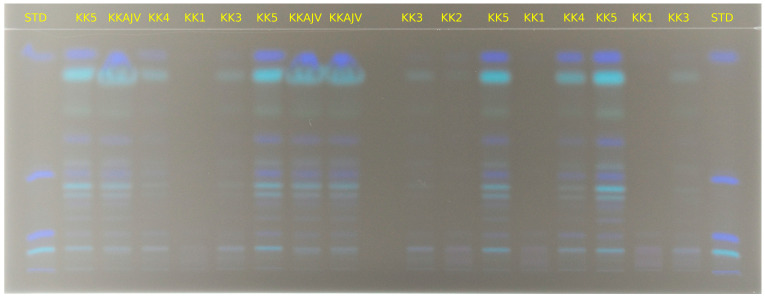
Carotenoid profile of investigated paprika and Ajvar samples obtained by HPTLC. STD—standards (also see Figure 4); KK1—Kurtovska kapija stage 1; KK2—Kurtovska kapija stage 2; KK3—Kurtovska kapija stage 3; KK4—Kurtovska kapija stage 4; KK5—Kurtovska kapija stage 5; KKAJV—Kurtovska kapija Ajvar.

**Figure 6:**
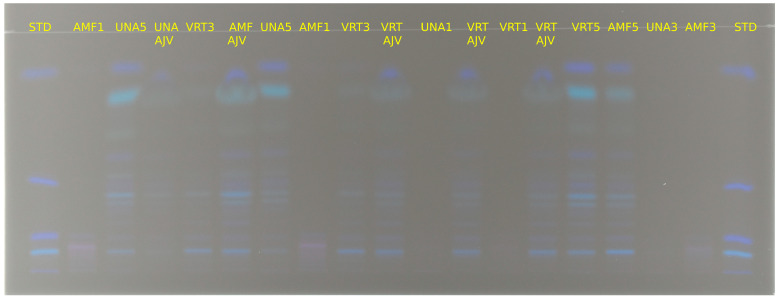
Carotenoid profile of investigated paprika and Ajvar samples obtained by HPTLC. STD—standards (also see Figure 4); UNA1—Una stage 1; UNA3—Una stage 3; UNA5—Una stage 5; UNAAJV—Una Ajvar; VRT1—Vrtka stage 1; VRT3—Vrtka stage 3; VRT5—Vrtka stage 5; VRT AJV—Vrtka Ajvar; AMF1—Amfora stage 1; AMF3—Amfora stage 3; AMF5—Amfora stage 5; AMFAJV—Amfora Ajvar.

Results of chromatographic separation of investigated samples are displayed on Figure 5 and Figure 6. It can be concluded that carotenoids are present in all samples, and depending on genotype and stage of maturation, bands are more or less intensive, which corresponds to the increasing or decreasing of the amount of a particular carotenoid.

**Figure A5:**
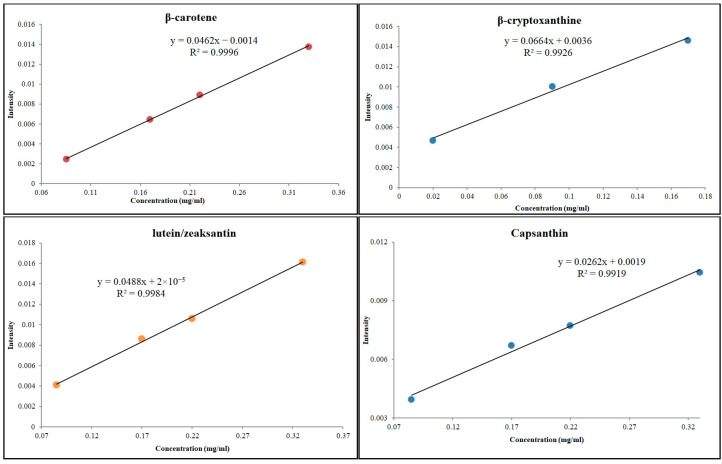
Calibration curves of analytical standards obtained using the HPTLC analytical method.

Quantification of the listed carotenoids was performed based on a calibration curve formed for concentrations ranging from 0.085 to 0.033 mg/mL (Figure A5). The concentration range for forming standard curves for individual carotenoids was determined through preliminary tests on a randomly selected extract sample. Table 2 shows the amount of individual and total carotenoids (g/100 g DM) in the extracts of the studied paprika genotypes and their traditional product, Ajvar. It also presents the percentage change in carotenoid levels during fruit development, both relative to the initial stage and the previous ripening stage.

The HPTLC analysis results display that capsanthin content ranges from 0.0708 g/100 g (in the Amfora genotype at stage 3) to 0.51 g/100 g (in the Vrtka genotype at stage 5). Its amount decreases in Ajvar across all studied genotypes. When it comes to lutein/zeaxanthin, the results generally show an upward trend, with the highest concentration in the final ripening stage (stage 5). Lutein/zeaxanthin levels range from 0.01 g/100 g (in the Una genotype at stage 1) to 1.62 g/100 g (in the Vrtka genotype at stage 5). In Ajvar, the lutein/zeaxanthin content decreases across all genotypes compared to values recorded in stage 5. It should be noted that the decrease (in %) in total investigated carotenoid content (TICC), which represents the sum of all investigated compounds in the traditional product (Ajvar) compared to stage 5 (final stage of maturation), was the smallest for the Kurtovska kapija genotype (1.9%), Vrtka (16.28%), and Amfora (19.53%), while the largest decrease was recorded for the Una genotype (66.98%). These results indicate that Ajvar should be made from the Kurtovska kapija genotype to retain a maximal amount of these valuable bioactive compounds.

The β-cryptoxanthin analysis revealed that this carotenoid is first detected in stage 5 across all studied genotypes, with amounts ranging from 0.013 g/100 g (in the Una genotype) to 0.08 g/100 g (in the Vrtka genotype). Amount of β-cryptoxanthin decrease in Ajvar compared to stage 5 across all genotypes.

The β-carotene content increases with ripening, reaching its highest level in the last stage (stage 5), with amounts ranging from 0.0900 g/100 g (in the Kurtovska kapija genotype at stage 1) to 0.99 g/100 g (in the same genotype at stage 5). This carotenoid’s levels also decrease in Ajvar for all genotypes compared to stage 5.

Examining the carotenoid profile of the Kurtovska kapija genotype reveals that capsanthin content remains constant in the first four ripening stages, with a marked increase to 0.36 g/100 g in the physiological maturity stage. Lutein/zeaxanthin content was elevated several times, from the technological maturity stage (stage 1) (0.03344 g/100 g) to the physiological maturity stage, labeled as stage 5 (0.15 g/100 g). The β-carotene content also rises more than tenfold with ripening, reaching 0.99 g/100 g in physiological maturity, while β-cryptoxanthin is absent in the first four stages and first appears in stage 5 at 0.05 g/100 g. In Ajvar, capsanthin, lutein/zeaxanthin, and β-cryptoxanthin levels significantly decrease compared to the physiological maturity stage (stage 5), while β-carotene levels are not statistically different from stage 5 (Table 2).

The carotenoid profile of the Amfora genotype is characterized by the complete absence of capsanthin and β-cryptoxanthin at technological maturity (stage 1), while lutein/zeaxanthin and β-carotene levels are 0.07 and 0.10 g/100 g, respectively. Comparing the technological and physiological maturity stages reveals a statistically significant increase in all tested carotenoids. Carotenoid analysis in Ajvar extracts showed a statistically significant decrease in all tested carotenoids (Table 2) except for β-carotene, where the observed reduction in content was not statistically significant.

The carotenoid profile of the Una genotype is characterized by the absence of capsanthin, β-cryptoxanthin, and β-carotene until physiological maturity (stage 5), when they accumulate in significant quantities of carotenoids (capsanthin 0.11 g/100 g; lutein/zeaxanthin 0.08 g/100 g; β-cryptoxanthin 0.013 g/100 g; β-carotene 0.17 g/100 g). However, after thermal processing (making Ajvar), β-carotene and lutein/zeaxanthin levels decrease significantly, while capsanthin and β-cryptoxanthin are undetectable (Table 2).

The carotenoid profile of the Vrtka genotype is characterized by the absence of capsanthin, β-cryptoxanthin, and β-carotene in the technological ripening stage (stage 1), while lutein/zeaxanthin is present at 0.03 g/100 g. During ripening, all tested carotenoids increase, but their levels partially reduce during processing (Table 2). Capsanthin content in stage 3 is 0.26 g/100 g, which significantly increases to 0.51 g/100 g in stage 5. In contrast, during Ajvar production, its content significantly decreases to 0.46 g/100 g. Lutein/zeaxanthin levels range from 0.03 g/100 g in stage 1 to 0.16 g/100 g in stage 5, with no significant decrease in Ajvar, remaining at 0.16 g/100 g. The β-cryptoxanthin first appears in stage 5 in a concentration of 0.08 g/100 g, while its amount in Ajvar is 0.07 g/100 g. Furthermore, results displayed that β-carotene first appears in stage 3 with a concentration of 0.12 g/100 g and significantly increases to 0.72 g/100 g in stage 5. In Ajvar made from this genotype, β-carotene levels decrease compared to stage 5 and amount to 0.55 g/100 g (Table 2).

The total investigated carotenoid content (TICC) (i.e., capsanthin + lutein/zeaxanthin + β-cryptoxanthin + β-carotene) (Table 2) generally followed an increasing trend with ripening across all genotypes, while the decrease was observed in Ajvar. The highest total carotenoid content was recorded, as expected, at full ripeness (stage 5) for all tested genotypes. In the Kurtovska kapija genotype, the highest total carotenoid content ranged from 0.25 g/100 g in stage 1 to 1.55 g/100 g in stage 5. In the Amfora genotype, levels ranged from 0.1696 g/100 g in stage 1 to 1.28 g/100 g in stage 5. The Vrtka genotype exhibited the highest percentage increase in TICC at full ripeness (stage 5) compared to the initial ripening stage (stage 1). Specifically, TICC in the initial ripening stage was 0.12 g/100 g, while in the final ripening stage (stage 5) it increased by over 5000%, reaching 1.47 g/100 g. The Una genotype is characterized by the lowest TICC in the initial ripening stage compared to other genotypes and amounts to 0.01 g/100 g and similarly shows the lowest total carotenoid content in the final ripening stage compared to all other studied genotypes (0.38 g/100 g).

The percentage decrease in TICC in the traditional product (Ajvar) compared to stage 5 was the smallest for the Kurtovska kapija genotype (1.9%), followed by Vrtka (16.28%) and Amfora (19.53%), while the largest decrease was recorded for the Una genotype (66.98%) (Table 2). These results indicate that Ajvar should ideally be made from the Kurtovska kapija genotype to preserve a maximal possible amount of these valuable bioactive compounds. It should also be noted that the percentage decrease in total carotenoids in Ajvar compared to stage 5 is in a strong negative correlation with the β-carotene content in stage 5 (r = 0.9536, r^2^ = 0.9094) (Figure A5). More precisely, the genotype that had a higher content of β-carotene in stage 5 also had a moderate decrease in total carotenoids in Ajvar.

### 3.2. Total Carotenoids Analysis

Regarding total carotenoids content (TCC) measured spectrophotometrically, the results showed an increase in all examined genotypes during the ripening (Table 3). For example, in the Vrtka genotype, the elevation of total carotenoids from the initial to the final ripening stage was 4713.23%. In the Una genotype, it was 1332.10%; in the Amfora genotype, 587.83%; and in the Kurtovska kapija genotype, it was 396.13% (Table 3). In the Kurtovska kapija genotype specifically, the smallest percentage increase in total carotenoids, as expected, was observed between stages 2 and 3.

The highest TCC was recorded at full ripeness across all genotypes studied, with the highest values observed in the Vrtka (1502.21 mg/100 g) and Kurtovska kapija (1498.21 ± 60.89 mg/100 g) genotypes. The lowest values were recorded at the initial ripening stage (stage 1) in all genotypes, with the lowest value noted in the Una genotype (20.22 mg/100 g) (Table 3). Analysis of variance (ANOVA) showed that the difference in TCC between stages 3 and 5 was statistically significant across all genotypes studied. Additionally, the TCC of Ajvar was significantly lower compared to the full ripeness stage for all tested samples.

**Figure A6:**
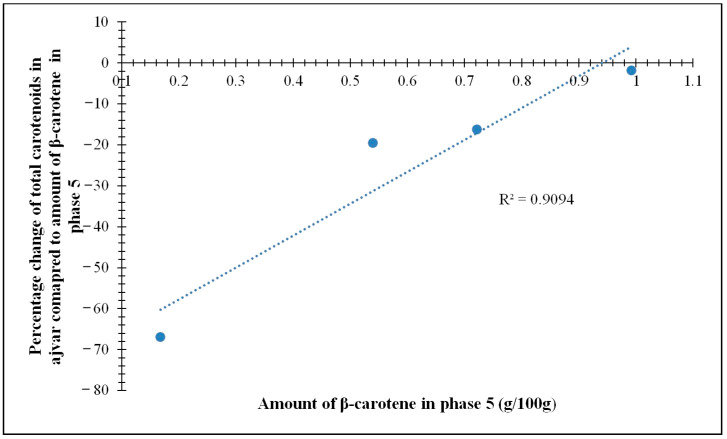
Correlation between change (in %) of total carotenoids in Ajvar compared to stage 5 and amount of β-carotene in stage 5.

It is important to note that the total carotenoid content measured by the HPTLC method is in a very strong positive correlation (r = 0.9930, r^2^ = 0.9863) with the total carotenoids measured spectrophotometrically (Figure A6).

### 3.3. Quantitative Analysis of Total Carotenoids in Paprika Fruit Using Raman Spectroscopy

**Figure 7:**
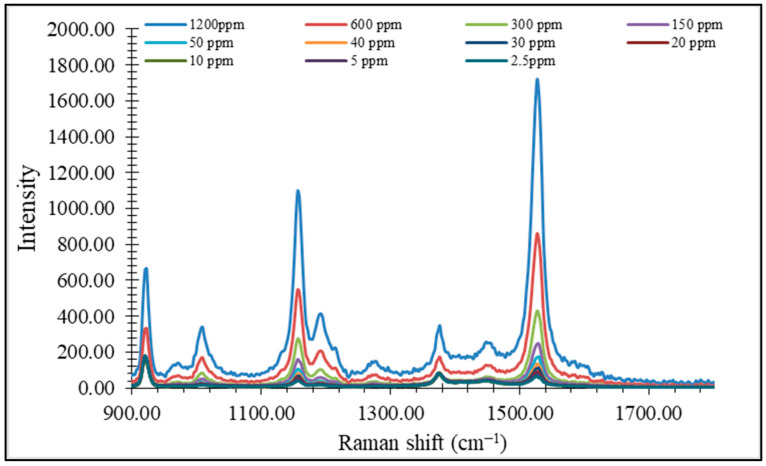
Raman spectra of β-carotene standard at different concentrations.

Figure 7 represents spectra of β-carotene in different concentrations. Bands around 1003, 1154, and 1524 cm^−1^ are crucial for identifying carotenoids [17]. The band around 1003 cm^−1^ corresponds to CH_3_ in-plane rocking vibrations originating from the conjugated polyene chain of carotenoids [18], while the band at 1154 cm^−1^ is because of C-C stretching vibrations in the carotenoid main chain. However, the band at 1524 cm^−1^ can be attributed to C-C stretching vibration carotenoids [19] and additionally to carotenoids with nine conjugated double bonds [20] (i.e., β-carotene).

**Figure 8:**
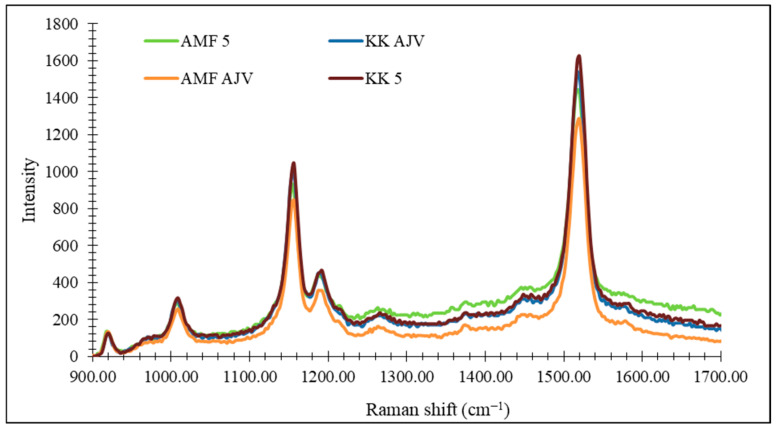
Averaged Raman spectra of investigated samples. KK 5—Kurtovska kapija stage 5; KK AJV—Kurtovska kapija Ajvar; AMF 5—Amfora stage 5; AMF AJV—Amfora Ajvar.

The chemometric modeling of Raman spectra was applied in an attempt to quantify total carotenoids, where the corrective factor was the total carotenoid content determined by the HPTLC method and spectrophotometrically. The genotypes used as model samples were Kurtovska kapija and Amfora in the fully ripe stage, as well as the corresponding Ajvar. To obtain the most reliable results, Raman spectroscopy was used to record identical extracts that were previously prepared for HPTLC analysis and total carotenoid analysis. The spectra of the investigated samples are displayed in Figure 8.

For the purpose of forming a quantification (predictive) model, β-carotene was used as a standard to express total carotenoids. This choice was based on its dominance in paprika fruit, as well as the ability to compare results with scientific literature where β-carotene is commonly used as a measure of total carotenoids [21].

Quantification of this compound was performed based on a calibration curve constructed within a concentration range of β-carotene from 2.5 ppm to 1320 ppm (Figure 7).

Preprocessing of the spectra included only baseline correction and smoothing to avoid distorting the intensity ratio of the bands, after which a Multivariate Linear Regression model was formed.

**Figure A7:**
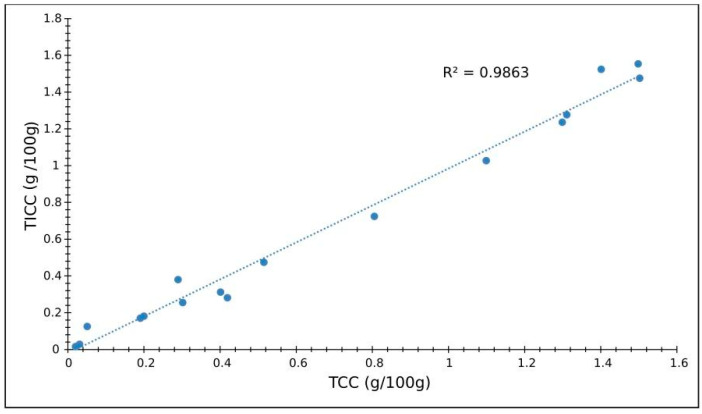
Dependence between total carotenoids measured spectrophotometrically and total examined carotenoids measured by the HPTLC method.

The results of the regression analysis are shown in Table 4 and Figure A7. The quantification results using the MLR method showed very high regression coefficients of determination (over 0.99).

### 3.4. Results of DPPH Test

The results of the DPPH• assay displayed an increase in radical inhibition during the maturation of paprika fruit in all investigated samples. Additionally, a tendency of slight decrease in inhibition of Ajvar extracts in comparison to the physiological maturation stage (stage 5) was observed in all genotypes (Table 5).

The genotype Kurtovska kapija (1.50 μmol equivalent TEAC/g DM) and Vrtka (1.49 μmol equivalent TEAC/g SM) showed the highest ability to quench DPPH•, followed by Amfora (1.39 μmol equivalent TEAC/g DM), while the lowest value was recorded in the genotype Una (1.18 μmol TEAC equivalents/g DM), which is in accordance with the results obtained by the HPTLC method of total carotenoids and spectrophotometrically.

The recorded results of DPPH radical inhibition are correlated positively with the results of total carotenoids.

On the other hand, the total carotenoid content in Ajvar slightly decreases compared to stage 5 in all tested genotypes, which is consistent with the observed trend in DPPH radical inhibition. DPPH radical inhibition decreased in Ajvar compared to stage 5 in the range from 6.78% to 16.03% (Table 5). When it comes to percentage changes in DPPH radical inhibition in Ajvar compared to the full maturity (stage 5), it was noted that the highest percentage decrease was recorded in the genotype Vrtka (16.03%), followed by Kurtovska kapija (10.67%) and Amphora (10.27%), while the smallest decline was recorded in the Una genotype (6.78%).

Table A1 presents the correlation analysis results, indicating statistically significant correlations between antioxidant activity (DPPH assay) and total carotenoid content (measured spectrophotometrically and via HPTLC) across all tested genotypes, except for the Una genotype.

### 3.5. Results of CUPRAC Test

The results of the CUPRAC test also showed that the values increased with the ripening of the paprika fruit (from technological to physiological maturity), while in Ajvar, the antioxidant potential decreased slightly.

The highest value in the stage of full maturity (stage 5) was recorded in the genotype Amfora (stage 5) and was 0.305 mg GA/g DM, followed by Kurtovska kapija (0.297 mg GA/g DM) and Una (0.249 mg GA/g DM), while the lowest value was recorded in the genotype Vrtka (0.225 mg GA/g DM) (Table 6). The lowest value of the CUPRAC test was displayed in the Vrtka genotype in the stage of technological maturity (0.178 mg GA/g DM) (stage 1) (Table 6).

Regarding the percentage change in antioxidant capacity in stage 5 compared to the initial ripening stage (stage 1), the largest increase was recorded in the genotype Amfora (64.43%), while the smallest was recorded in the genotype Vrtka and amounted to 26.40% (Table 6).

The results displayed that the reduction in carotenoids in Ajvar compared to the full maturity stage (stage 5) is the smallest in the Kurtovska kapija genotype, with only 2.12% lower antioxidant capacity. Also, the Vrtka genotype showed low values, and it amounted to 8.89%. The greatest decrease in antioxidative capacity during paprika processing (making Ajvar) was recorded in Amfora (20.38%) and Una (20.48%) genotypes (Table 6). These results are in agreement with the results of the percentage change of total carotenoids measured spectrophotometrically. More precisely, Kurtovska kapija and Vrtka genotypes showed the lowest percentage change in total carotenoids during the technological process of making the final product.

### 3.6. Results of the FRP Test

The results of FRP analysis show that the antioxidant capacity increases with paprika ripening in all genotypes, while in the product (Ajvar) it does not change significantly in relation to the values in the final physiological stage of ripening. The highest values of antioxidant capacity were obtained by the genotypes Kurtovska kapija (25.66 mgGA/g DM) and Amfora (24.23 mgGA/g DM), while the lowest values were recorded in the genotypes Una (15.81 mgGA/g DM) and Vrtka (15.22 mgGA/g DM) (Table 7). When it comes to the reduction capacity of Fe^3+^ ions in Ajvar, the smallest percentage decrease compared to the final stage of ripening was observed in the Kurtovska kapija genotype (2.1%), followed by the Amfora genotype (4.75%) and Vrtka (8.89%), while the Una genotype showed a slight increase of 2.02%, which was not statistically significant (Table 7).

### 3.7. Bioavailability of Carotenoids from Paprika Fruit and Ajvar of Genotype Una

The total carotenoid content (measured spectrophotometrically) in lyophilized samples of the Una genotype at different ripening stages as well as in Ajvar after in vitro gastrointestinal digestion are shown in Table 8. Accordingly, total carotenoid content after digestion was 7.52 mg/100 g DM in the paprika sample at stage 1, 7.62 mg/100 g DM in the paprika sample at stage 3, 16.19 mg/100 g DM in the paprika sample at stage 5, and 66.86 mg/100 g DM in Ajvar. As can be seen, the total carotenoid content in paprika fruit samples at successive developmental stages, especially at full maturity, was significantly lower compared to Ajvar.

## 4. Discussion

### 4.1. Influence of Genotype on Carotenoid Composition and Stability During Thermal Processing

The results obtained using the HPTLC method showed that within the investigated genotypes, Kurtovska kapija and the indigenous genotype Vrtka have the highest amounts of total investigated carotenoids (1498 mg/100 g and 1502 mg/100 g, respectively), while genotype Una contains the smallest amount of total investigated carotenoids (289 mg/100 g). Similarly, in the study by [22], the total carotenoid content in the final ripening stage in certain genotypes could differ significantly, i.e., could reach a maximum of about 130 mg/100 g (genotype Anupan), while in others it could be below 11 mg/100 g (genotype Flamingo). All of this leads to the conclusion that the carotenoid content is a genotype-dependent trait [23].

Topaz et al. [24] investigated the carotenoid composition in five sweet paprika genotypes at the physiological maturity stage, and results revealed that the predominant carotenoids at this stage were capsanthin, lutein/zeaxanthin, β-carotene, and β-cryptoxanthin. Moreover, the total carotenoid content varied between 809.2 and 1340 mg/kg DM. These findings align with our study, confirming that carotenoid levels are genotype-dependent. The results of Rodríguez-Rodríguez et al. [25] also show that the predominant carotenoids in the stage of physiological maturity of sweet paprika are precisely capsanthin, lutein/zeaxanthin, β-carotene, and β-cryptoxanthin, and that violaxanthin also occurs as a dominant carotenoid. In our study, when it comes to qualitative carotenoid composition, the amount of capsanthin was in the range of 0.11–0.51 g/100 g DM; lutein/zeaxanthin was in the range of 0.08–0.16 g/100 g DM; β-cryptoxanthin was in the range of 0.01–0.8 g/100 g DM; and β-carotene was in the range of 0.17–0.99 g/100 g DM, which is in agreement with the literature data (Rodríguez-Rodríguez et al. [25] and Deli et al. [26]). According to the results of Deli et al. (2001) [26], the total carotenoid content significantly increases as the fruit ripens, ranging from 19.60 mg/100 g DM at the technological ripeness stage to 1297.12 mg/100 g DM at full ripeness, which is also similar to the results obtained in this paper. The biosynthesis of carotenoids in all genotypes in our study, except for the Una genotype, begins in the early stages of growth and development, leading to increased accumulation of the investigated carotenoids, primarily β-carotene, during fruit ripening. As a result of this late accumulation of carotenoids in the Una genotype, the final amount of investigated carotenoids in fruits is significantly lower. Deli et al. [26] have conducted a comprehensive analysis of carotenoids in sweet paprika at six developmental stages. The results showed that 34 different carotenoids were detected, the most dominant of which were lutein, β-carotene, capsanthin, zeaxanthin, and β-cryptoxanthin.

It can be concluded, based on the previous studies, that the amount of carotenoids largely depends on the genotype, growing conditions, method of extraction, as well as the instrument used to analyze it [23,26]. The results of our study indicate a consistent pattern in carotenoid concentrations across all examined genotypes at the final stage of ripening (stage 5). Their presence is distributed as follows, starting from the most abundant to the least abundant: β-carotene, capsanthin, lutein/zeaxanthin, and β-cryptoxanthin. When it comes to β-carotene content, its high presence in the final stage of ripening in all genotypes can be explained by the fact that at the moment of fruit picking, biosynthesis was interrupted and that biosynthesis would potentially have continued in the direction of capsanthin formation if the fruit had remained on the plant, which will be the subject of our future research in terms of confirming or rejecting this assumption. Another explanation is that it is a genotype characteristic [25,27]. According to Hornero-Méndez et al. [28], during the ripening of paprika fruits, the amount of lutein and β-carotene decreases, while the amount of β-cryptoxanthin and capsanthin increases. However, the biosynthesis of carotenoids, as well as their amount in the fruit, is influenced by a large number of factors, such as higher or lower expression of genes that control carotenogenesis, effects of physiological and morphological characteristics of the genotype, growing conditions, etc. [28]. Considering that, in certain genotypes, it can happen that the amount of β-carotene is more dominant than capsanthin [29]. Deli et al. [30] reported that carotenoids with nine conjugated double bonds occurring in genotype red paprika are lutein, β-carotene, zeaxanthin, β-cryptoxanthin, and capsanthin, and their amount changes with ripening, which was also confirmed in this study.

The traditional product Ajvar contains slightly lower total carotenoid levels compared to the final ripening stage, with an average percentage decrease of about 6.48% for Kurtovska kapija, 13.52% for Vrtka, 16.14% for Amfora, and about 82% for Una. This result is consistent with the literature [31], which suggests that some carotenoids, such as β-carotene, do not degrade significantly during thermal processing. The thermolabile nature of carotenoids, including β-carotene, is well known; however, scientific studies have shown that the stability of β-carotene at high temperatures increases in the presence of oil (i.e., unsaturated fatty acids) [32,33]. The β-carotene is a lipophilic compound that dissolves in oil, which likely results in being encapsulated within the oil droplet [34]. Its degradation is prevented by the presence of the oil matrix, as was the case with Ajvar. The oil droplet in Ajvar probably acts as a barrier, physically separating β-carotene from the external environment, including direct exposure to high temperatures. Moreover, the oil droplet absorbs and distributes heat evenly, preventing localized heat damage, and this environment protects β-carotene from oxidative degradation [26,35,36].

### 4.2. Quantification of Carotenoids Using Raman Spectroscopy

Thus far, only a limited number of studies exploring the application of Raman spectroscopy in the quantification of total carotenoids have been published. Sebben et al. [37] examined the applicability of Raman spectroscopy and PLSR (Partial Least Square Regression) in the quantification of carotenoids in sweet potato and reported high R^2^ values ranging from 0.90 to 0.99, depending on the spectral range and method of sample processing. However, results of Hara et al. (2018) [38] on tomato displayed significantly lower R^2^ values, and it depended on the exposure time of the laser, while the application of ANN (Artificial Neural Networks) to quantify carotenoids in tomato resulted in R^2^ values around 0.9 [39]. Killeen et al. (2013) [40] investigated the possibility of Raman spectroscopy in the quantification of carotenoids in 31 different carrot samples, achieving R^2^ values of 0.88 using MLR (Multiple Linear Regression). Baranska et al. (2013) [17] utilized Raman spectroscopy coupled with PLSR to quantify lycopene and β-carotene, yielding R^2^ values of 0.91 and 0.89, respectively, while Lawaetz et al. (2016) [41] demonstrated that Raman spectroscopy in combination with PLSR could quantify carotenoids in carrot (*Daucus carota*), achieving an R^2^ value of 0.86.

The results presented in the aforementioned studies are consistent with those obtained in our study, especially in terms of validating the findings using standard analytical methods for carotenoid determination.

### 4.3. Influence of Genotype, Ripening, and Thermal Processing on Antioxidant Capacity

All conducted antioxidant tests showed a trend of increasing values with fruit ripening. In the case of DPPH, obtained results are in accordance with previously published research results [42,43,44,45]. Sun et al. (2007) [45] examined the ability to inhibit DPPH radicals during paprika ripening, and the results showed that the inhibition increased with ripening. On the other hand, Ghasemnezhad et al. (2011) [44] examined the differences in the capture of DPPH radicals in two stages of maturation (technological and physiological) and concluded that the ability to capture the tested radical is greater in the physiological stage of maturation. Fitriansyah et al. (2018) [46] examined the relationship (correlations) between the amount of carotenoids in the extract of the *Phyllanthus emblica* plant and the inhibition of DPPH radicals and concluded that there is a strong positive correlation (r = 0.97). These statements are consistent with our results and indicate the importance of carotenoids in neutralizing harmful oxygen species. The results of the CUPRAC test are positively correlated with the values obtained in the DPPH assay, which is expected considering that both tests are designed to measure the ability of substances to act as antioxidants. The difference is in the mechanism (principles) of the methods themselves, but the goal of the methods is the same. It is also known that the results of these two tests show the same trend [47]. When it comes to FRP analysis, the literature data, in most cases, show a similar trend, although some genotypes exhibit an opposite trend [22,48]. Ajvar had a lower inhibitory capacity compared to the corresponding paprika genotype in the final ripening stage.

### 4.4. Bioavailability of Total Carotenoids in Paprika Samples at Various Ripening Stages and in Ajvar

Under simulated in vitro digestion conditions, extracts from the Una genotype at different stages of ripening showed varying bioavailability. Differences in the bioavailability of total carotenoids at different stages of maturity may be the result of the presence of certain dominant carotenoids during fruit development, as well as their sensitivity to different conditions to which they are exposed during gastrointestinal digestion. During the passage of carotenoid-rich food through the gastrointestinal tract, it is exposed to different environments and conditions [49]. Each of these gastrointestinal regions is specialized for specific mechanical, chemical, and enzymatic processes, including the composition of proteins, salts, different pH, and especially the type of digestive enzymes [50]. During the oral digestion stage in our experiment, the rehydrated lyophilized paprika samples were mixed with simulated salivary fluid (SSF). Following this, in the gastric stage (stomach), the food/nutrient matrix was decomposed by simulated gastric fluid (SGF), resulting in the release of carotenoids. In the intestinal stage (small intestine), the released carotenoids were mixed with a simulated intestinal fluid (SIF), which is designed to imitate the conditions in the small intestine where carotenoids are absorbed through the microvilli of enterocytes, i.e., epithelial cells of the inner side of the intestinal wall.

It is well known that lutein and zeaxanthin are stable in a neutral to slightly acidic environment (pH 6–7) [51] but degrade in alkaline environments (especially over 8) [52]. At a high pH value, the isomerization of lutein and zeaxanthin occurs, which leads to a decrease in their stability as well as a decrease in coloration, consequently affecting their biological properties. High pH values are not characteristic of any compartment of the gastrointestinal tract, so a more efficient absorption of lutein/zeaxanthin could be expected. On the other hand, β-carotene is a carotenoid that is also relatively stable at pH from slightly acidic to neutral (pH~6–7), but at a lower pH value, i.e., in an acidic environment, it is subject to chemical degradation, which leads to a loss of color and a decrease in the nutritional value of food in conditions of reduced pH [53,54]. Given that phases 1 and 3 are rich in carotenoids that are more stable in terms of pH changes in the oral (pH~6–7), gastric (pH~1–5) and intestinal digestion (pH~6–7) phases (lutein and zeaxanthin), this can explain the higher percentage of bioavailability compared to the full maturity stage (stage 5), which is rich in less stable carotenoids sensitive to the pH value changes, and especially to the conditions of an acidic environment (β-carotene and capsanthin).

However, the amount of total carotenoids in Ajvar after digestion is higher compared to the lyophilisate of ripe paprika fruit (i.e., the material from which Ajvar is made). Ajvar is a product that is rich in sunflower oil and therefore represents a lipophilic matrix. During digestion, in the gastric phase, oil is broken down by the pancreatic lipase, where glycerol and fatty acids are obtained as a product. Glycerol, together with bile salts from the gastric phase, surrounds the carotenoid molecules and thus represents a protection against further degradation.

The low bioavailability of carotenoids and their esters after digestion was also described by [55] for the plant *Pouteria lucuma*. However, in this work, the bioavailability of individual compounds was examined with the help of more detailed chromatographic analysis. Unlike paprika, where a reduced bioavailability of total carotenoids was observed, in the Ajvar sample, the bioavailability of total carotenoids was higher, even by 130.81%. However, if we consider that Ajvar contains oil, it can be assumed that the oil droplets “encapsulate” carotenoids, thereby protecting them from degradation and improving their bioavailability after digestion. Under the influence of lipases (from pancreatin), oils are converted to fatty acids and glycerides, which together with bile salts form mixed micelles [56], which surround carotenoids and increase their bioavailability. Otherwise, it is well known that the adoption, i.e., absorption of carotenoids in the intestinal tract, increases significantly in the presence of lipids, i.e., in a lipophilic matrix [57,58,59]. However, this research is based on the results of spectrophotometric analysis, so additional analyses (using Raman spectroscopy and chromatographic techniques) are desirable in order to obtain more reliable results for the bioavailability of individual carotenoids.

It should also be noted that the bioavailability of hydrophobic bioactive compounds (including carotenoids) is higher when consumed in food rather than in supplements due to the matrix in which they are naturally embedded [60].

## 5. Conclusions

The application of various analytical methods in analyzing carotenoids in paprika fruits, standard ones like HPTLC as well as innovative ones like Raman spectroscopy, confirmed the presence of different groups of carotenoids, whose concentration and composition change during fruit ripening, gradually increasing until the stage of full maturity. The relative ratio between different types of carotenoid compounds was comparable, but the total amount of carotenoids differed significantly between the investigated genotypes. The total amount of carotenoids in Ajvar was lower compared to thermally untreated samples, probably due to carotenoid instability and their thermal degradation during preparing this traditional product, but the bioavailability of carotenoids in Ajvar was much higher compared to dry paprika fruit samples. The highest bioavailability was recorded in Ajvar, likely due to its “encapsulation” in oil droplets, i.e., the formation of oil micelles, as well as the additional encirclement by bile salts and glycerol, which protected the carotenoids from further degradation. The antioxidant capacity of the extracts from the investigated paprika genotypes was directly dependent on the amount of total carotenoids, confirming their biological significance as nutrients with a high capacity for neutralizing free oxygen radicals. Raman spectroscopy has shown potential in quantifying total carotenoids in the paprika extract of the investigated genotypes as well as in the Ajvar extract, using a Multivariate Linear Regression model. It can be expected that Raman spectroscopy will increasingly be applied in the determination and quantification of important nutrients and bioactive plant molecules in various biological raw materials, including very complex matrix systems such as food samples and food products. Future research will also be directed towards applying Raman spectroscopy combined with artificial intelligence and robotics to enable in situ rapid and efficient determination of the quality and safety of raw materials and food in real time. When it comes to quantification, future research should focus on separating carotenoids from mixtures and on simultaneous quantification of a greater number of dominant carotenoids from the sample. Additionally, it is necessary to ensure the reproducibility of the model by training it with a training set so that it can continuously improve the existing model with new data.

## Figures and Tables

**Table 1:** Composition of fluids used in in vitro digestion.

Chemicals	SSF	SGF	SIF
	pH 7.0	pH 3.0	pH 7.0
	mmol∙L^−1^	mmol∙L^−1^	mmol∙L^−1^
KCl	15.1	6.9	6.8
KH_2_PO_4_	3.7	0.9	0.8
NaHCO_3_	13.6	25	85
NaCl	-	47.2	38.4
MgCl_2_·6H_2_O	0.15	0.1	0.33
(NH_4_)_2_CO_3_	0.06	0.5	-
NaOH	-	-	8.4
HCl	1.1	15.6	-
CaCl_2_·2H_2_O	0.75	0.075	0.3

SSF—simulated salivary fluid; SGT—simulated gastric fluid; SIF—simulated intestinal fluid.

**Table 2:** Quantification of carotenoids using high-performance thin-layer chromatography (HPTLC).

Genotype	Ripening Stage	Investigated Carotenoids				TICC (Capsanthin + Lutein/Zeaxanthin + β-Cryptoxanthin + β-Carotene (g/100 g % DM)	Change in the TICC Through Ripening Stages and in the Product Compared to the Initial Stage—Stage 1 (%)	Change in the TICC Through Ripening Stages and in the Product Compared to the Previous Stage (%)
		Capsanthin (g/100 g DM ± SD)	Lutein/Zeaksantin (g/100 g DM ± SD)	β-Cryptoxanthin (g/100 g DM ± SD)	β-Carotene (g/100 g DM ± SD)			
Kurtovska kapija	1	0.13 ± 0.06 ^a^	0.03 ± 0.01 ^a,B^	n.d.	0.09 ± 0.01 ^a,A^	0.25	0.00	0.00
	2	0.13 ± 0.003 ^a^	0.05 ± 0.003 ^b^	n.d.	0.13 ± 0.01 ^bc^	0.31	22.26	22.26
	3	0.13 ± 0.04 ^a,A^	0.04 ± 0.02 ^a,C^	n.d.	0.12 ± 0.01 ^b,B^	0.28	10.37	−9.73
	4	0.14 ± 0.03 ^a^	0.11 ± 0.03 ^c^	n.d.	0.47 ± 0.11 ^d^	0.72	183.94	157.27
	5	0.36 ± 0.08 ^c,B^	0.15 ± 0.04 ^e,B^	0.05 ± 0.01 ^b,A^	0.99 ± 0.14 ^e,D^	1.55	509.62	114.70
	Ajvar	0.30 ± 0.08 ^b,A^	0.14 ± 0.03 ^d,B^	0.04 ± 0.01 ^a,B^	0.98 ± 0.12 ^e,C^	1.52	498.04	−1.90
Amfora	1	n.d.	0.07 ± 0.009 ^b,C^	n.d.	0.10 ± 0.01 ^b,A^	0.17	0.00	0.00
	3	0.07 ± 0.0 08 ^a,B^	0.02 ± 0.006 ^a,B^	n.d.	0.09 ± 0.008 ^a,A^	0.18	6.49	6.49
	5	0.50 ± 0.09 ^c,C^	0.15 ± 0.04 ^d,B^	0.07 ± 0.03 ^b,B^	0.54 ± 0.14 ^c,B^	1.28	652.42	606.59
	Ajvar	0.35 ± 0.13 ^b,A^	0.12 ± 0.04 ^c,B^	0.03 ± 0.01 ^a,A^	0.53 ± 0.04 ^c,B^	1.03	505.48	−19.53
Una	1	n.d.	0.01 ± 0.001 ^a,A^	n.d.	n.d.	0.01	0.00	0.00
	3	n.d.	0.02 ± 0.005 ^b,A^	n.d.	n.d.	0.01	6.58	6.58
	5	0.11 ± 0.07 ^A^	0.08 ± 0.05 ^d,A^	0.01 ± 0.007	0.17 ± 0.02 ^b,A^	0.38	2394.74	2240.74
	Ajvar	n.d.	0.033 ± 0.09 ^c,A^	n.d.	0.09 ± 0.008 ^a,A^	0.12	723.68	−66.98
Vrtka	1	n.d.	0.03 ± 0.03 ^a,A^	n.d.	n.d.	0.03	0.00	0.00
	3	0.26 ± 0.04 ^a,C^	0.09 ± 0.02 ^b,D^	n.d.	0.12 ± 0.01 ^a,B^	0.47	1580.50	1580.50
	5	0.51 ± 0.04 ^c,C^	0.16 ± 0.02 ^c,C^	0.08 ± 0.04 ^b,C^	0.72 ± 0.07 ^c,C^	1.47	5131.21	211.29
	Ajvar	0.456 ± 0.18 ^b,B^	0.16 ± 0.05 ^c,C^	0.071 ± 0.03 ^a,C^	0.55 ± 0.05 ^b,B^	1.23	4279.79	−16.28

Phases that share lowercase letters do not differ statistically significantly in terms of the amount of tested compounds. Phases that share the same capital letters do not differ statistically significantly in terms of the amount of tested compounds. Small letters are used to indicate statistically significant differences in the analyzed compounds between ripening stages within the same genotype. Capital letters are used to denote statistically significant differences in the analyzed compounds between genotypes in the same ripening stage. In all tests, a significance level of 5% was used. n.d.—not detected; SD—standard deviation; DM—dry mass.

**Table 3:** Analysis of total carotenoid content in paprika extracts and Ajvar.

Name of the Sample	Total Carotenoids mg/100 g	Change in TCC Through Ripening Stages and in the Product Compared to the Initial Stage—Stage 1 (%)	Change in TCC Through Ripening Stages and in the Product Compared to the Previous Stage (%)
Amfora stage 1	190.60 ± 3.23 ^a^	0.00	0.00
Amfora stage 3	200.21 ± 10.55 ^a^	5.04	5.04
Amfora stage 5	1311.01 ± 15.84 ^b^	587.83	554.82
Amfora Ajvar	1099.35 ± 15.62 ^c^	476.78	−16.14
Kurtovska kapija stage 1	301.98 ± 13.98 ^a^	0.00	0.00
Kurtovska kapija stage 2	401.56 ± 10.23 ^b^	32.98	32.98
Kurtovska kapija stage 3	419.31 ± 15.96 ^b^	38.85	4.42
Kurtovska kapija stage 4	805.45 ± 32.23 ^c^	166.72	92.09
Kurtovska kapija stage 5	1498.21 ± 60.89 ^d^	396.13	86.01
Kurtovska kapija Ajvar	1401.11 ± 59.01 ^e^	363.97	−6.48
Una stage 1	20.22 ± 1.63 ^a^	0.00	0.00
Una stage 3	22.01 ± 1.21 ^a^	8.85	8.85
Una stage 5	289.57 ± 14.58 ^b^	1332.10	1215.63
Una Ajvar	51.08 ± 2.66 ^c^	152.62	−82.36
Vrtka stage 1	31.21 ± 1.28 ^a^	0.00	0.00
Vrtka stage 3	515.05 ± 20.21 ^b^	1550.27	1550.27
Vrtka stage 5	1502.21 ± 70.74 ^d^	4713.23	191.66
Vrtka Ajvar	1299.09 ± 60.21 ^c^	4062.42	−13.52

Small letters are used to indicate statistically significant differences in the analyzed compounds between ripening stages within the same genotype. Phases that share lowercase letters do not differ statistically significantly in terms of the amount of tested compounds.

**Table 4:** Quantification of selected samples using Raman spectroscopy.

Selected Samples	TCC by RS Coupled with MLR Method (g/100 g DM)	TICC by HPTLC (g/100 g DM)	TCC Spectrophotometrically (g/100 g DM)
Kurtovska kapija stage 5	1.952	1.5527	1.4982
Kurtovska kapija Ajvar	1.899	1.5232	1.4011
Amfora stage 5	1.726	1.2791	1.3110
Amfora Ajvar	1.614	1.0269	1.0993

**Table 5:** Antioxidant capacity based on DPPH radical scavenging activity, expressed as μmol TEAC equivalent per gram of dry sample mass.

Name of the Sample	DPPH•	Change in the Inhibition of DPPH Radicals Through the Ripening Stages and in the Product in Relation to the Initial Stage—Stage 1 (%)	Change in DPPH Radical Inhibition Through the Ripening Stages and in the Product Compared to the Previous Stage (%)
	μmol/g TEAC		
Amfora stage 1	0.23 ± 0.03 ^a^	0.00	0.00
Amfora stage 3	0.61 ± 0.02 ^b^	165.22	165.22
Amfora stage 5	1.46 ± 0.19 ^d^	534.78	139.34
Amfora Ajvar	1.31 ± 0.15 ^c^	469.57	−10.27
Kurtovska kapija stage 1	0.21 ± 0.06 ^a^	0.00	0.00
Kurtovska kapija stage 2	0.40 ± 0.04 ^b^	90.48	90.48
Kurtovska kapija stage 3	0.91 ± 0.07 ^c^	333.33	127.50
Kurtovska kapija stage 4	1.04 ± 0.09 ^d^	395.24	14.29
Kurtovska kapija stage 5	1.50 ± 0.11 ^f^	614.29	44.23
Kurtovska kapija Ajvar	1.34 ± 0.15 ^e^	538.10	−10.67
Una stage 1	0.30 ± 0.04 ^a^	0.00	0.00
Una stage 3	0.89 ± 0.04 ^b^	196.67	196.67
Una stage 5	1.18 ± 0.02 ^c^	293.33	32.58
Una Ajvar	1.10 ± 0.02 ^d^	266.67	−6.78
Vrtka stage 1	0.21 ± 0.01 ^a^	0.00	0.00
Vrtka stage 3	0.46 ± 0.04 ^b^	223.91	223.91
Vrtka stage 5	1.49 ± 0.09 ^d^	184.78	−12.08
Vrtka Ajvar	1.31 ± 0.07 ^c^	139.13	−16.03

Small letters are used to indicate statistically significant differences in the analyzed compounds between ripening stages within the same genotype. Phases that share lowercase letters do not differ statistically significantly in terms of the amount of tested compounds.

**Table 6:** An analysis of antioxidant capacity using the CUPRAC method.

Name of the Sample	mg AsA/g DM	Change in CUPRAC Activity Through the Maturation Stages and in the Product in Relation to the Initial Stage—Stage 1 (%)	Change in CUPRAC Activity Through the Maturation Stages and in the Pro-Extract Compared to the Previous Stage (%)
Amfora stage 1	0.194 ± 0.03 ^a^	0.00	0.00
Amfora stage 3	0.205 ± 0.02 ^a^	5.67	5.67
Amfora stage 5	0.319 ± 0.02 ^c^	64.43	55.61
Amfora phase	0.254 ± 0.02 ^b^	30.93	−20.38
Kurtovska kapija stage 1	0.205 ± 0.02 ^a^	0.00	0.00
Kurtovska kapija stage 2	0.209 ± 0.02 ^a^	1.95	1.95
Kurtovska kapija stage 3	0.200 ± 0.02 ^a^	−2.44	−4.31
Kurtovska kapija stage 4	0.256 ± 0.02 ^b^	24.88	28.00
Kurtovska kapija stage 5	0.297 ± 0.02 ^c^	44.88	16.02
Kurtovska kapija Ajvar	0.291 ± 0.02 ^c^	41.95	−2.02
Una stage 1	0.185 ± 0.02 ^a^	0.00	0.00
Una stage 3	0.185 ± 0.02 ^a^	0.00	0.00
Una stage 5	0.249 ± 0.03 ^c^	34.59	34.59
Una Ajvar	0.198 ± 0.02 ^b^	7.03	−20.48
Vrtka faza 1	0.178 ± 0.02 ^a^	0.00	0.00
Vrtka faza 3	0.199 ± 0.02 ^b^	11.80	11.80
Vrtka faza 5	0.225 ± 0.02 ^c^	26.40	13.07
Vrtka Ajvar	0.205 ± 0.02 ^b^	15.17	−8.89

Small letters are used to indicate statistically significant differences in the analyzed compounds between ripening stages within the same genotype. Phases that share lowercase letters do not differ statistically significantly in terms of the amount of tested compounds.

**Table 7:** Analysis of antioxidant capacity using Fe^3+^ ion reduction ability.

Name of the Sample	mg AsA (GA)/g DM	Change in FRP Activity Through the Ripening Stages and in the Product in Relation to the Initial Stage—Stage 1 (%)	Change in FRP Activity Through the Ripening Stages and in the Product in Relation to the Previous Stage (%)
Amfora stage 1	12.36 ± 0.59 ^a^	0.00	0.00
Amfora stage 3	16.54 ± 0.73 ^b^	33.82	33.82
Amfora stage 5	24.23 ± 1.10 ^c^	96.04	46.49
Amfora Ajvar	23.08 ± 1.15 ^c^	86.73	−4.75
Kurtovska kapija stage 1	10.37 ± 0.52 ^a^	0.00	0.00
Kurtovska kapija stage 2	10.23 ± 0.51 ^a^	−1.35	−1.35
Kurtovska kapija stage 3	13.74 ± 0.72 ^b^	32.50	34.31
Kurtovska kapija stage 4	16.36 ± 0.75 ^c^	57.76	319.07
Kurtocska kapija stage 5	25.66 ± 1.30 ^d^	147.44	56.85
Kurtovska kapija Ajvar	25.12 ± 1.26 ^d^	142.24	−2.10
Una stage 1	9.33 ± 0.47 ^a^	0.00	0.00
Una stage 3	10.11 ± 0.51 ^a^	8.36	8.36
Una stage 5	15.81 ± 0.79 ^b^	69.45	56.38
Una Ajvar	16.13 ± 0.75 ^b^	72.88	2.02
Vrtka stage 1	10.11 ± 0.61 ^a^	0.00	0.00
Vrtka faza 3	13.16 ± 0.66 ^b^	11.80	11.80
Vrtka stage 5	15.22 ± 0.77 ^d^	26.40	13.07
Vrtka Ajvar	14.89 ± 0.80 ^c^	15.17	−8.89

Small letters are used to indicate statistically significant differences in the analyzed compounds between ripening stages within the same genotype. Phases that share lowercase letters do not differ statistically significantly in terms of the amount of tested compounds.

**Table 8:** Bioavailability of carotenoid compounds of paprika fruit extract before and after in vitro gastrointestinal digestion (results are presented as mg/100 g of dry matter).

Sample	Before In Vitro Digestion mg/100 g	After In Vitro Digestion mg/100 g	Bioavailability Relative to Initial Extract (%)
Una stage 1	20.22	7.52	37.19
Una stage 3	22.01	7.62	34.60
Una stage 5	289.57	16.19	5.59
Una Ajvar	51.08	66.82	130.81

**Figure A8:**
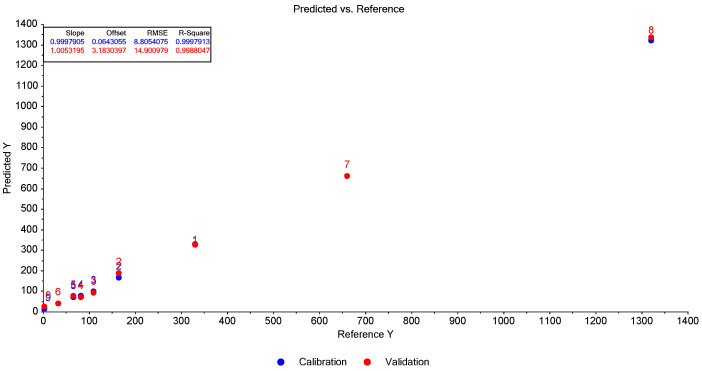
Dependence of the predicted reference values of the amount of β-carotene for the training and test set of data.

## Data Availability

The original contributions presented in the study are included in the article, further inquiries can be directed to the corresponding authors.

## Appendix A

**Table A1:** Pearson correlation coefficient between DPPH analysis values and total carotenoids measured spectrophotometrically.

Correlations Examined	Sorta	Amfora	Kurtovska Kapija	Una	Vrtka
Correlations between DPPH analysis results and total carotenoids measured spectrophotometrically	Pearson correlation coefficient	0.965 *	0.907 *	0.589	0.992 **
	*p*-value	0.035	0.013	0.411	0.008
Correlations between the results of the DPPH analysis and total carotenoids obtained by summing individual carotenoids measured by the HPTLC method	Pearson correlation coefficient	0.963 *	0.879 *	0.682	0.994 **
	*p*-value	0.037	0.021	0.318	0.006

* correlation is statistically significant (*p* < 0.05); ** correlation is statistically significant (*p* < 0.01).
